# GLP-1 and dual GIP/GLP-1 receptor agonists’ psychopharmacology and putative neuropsychiatric associated effects: a Bradford Hill–informed, systematic, evaluation

**DOI:** 10.1007/s11920-026-01709-w

**Published:** 2026-08-21

**Authors:** F Schifano, M A De Luca, S Bonaccorso, Davide Arillotta, G Floresta, G Martinotti, GD Papanti, J M Corkery, S Chiappini, A Guirguis

**Affiliations:** 1 Drug Misuse and Novel Psychoactive Substances Research Unit, University of Hertfordshire Medical School, Psychopharmacology, Hatfield, UK; 2 Department of Biomedical Sciences, University of Cagliari, Cagliari, Italy; 3 North London NHS Foundation Trust, St Pancras Hospital, London, UK; 4 Department of Neurosciences, Psychology, Drug Research, and Child Health, Section of Pharmacology and Toxicology, University of Florence, Florence, Italy; 5 Department of Drug and Health Sciences, University of Catania, Catania, Italy; 6 Department of Neurosciences, Imaging and Clinical Sciences, Università degli Studi G. D’Annunzio Chieti-Pescara, Chieti, Italy; 7 Tolmezzo Community Mental Health Centre, ASUFC Mental Health and Addiction Department, Tolmezzo, Italy; 8 UniCamillus International University of Medical Sciences, Rome, Italy; 9 University of Swansea Medical School, Swansea, UK

**Keywords:** GLP-1 receptor agonists, GLP-1 RAs, dual GIP/GLP-1 RAs, triple GIP/GLP-1/glucagon RAs, Semaglutide, Tirzepatide, Liraglutide, Retatrutide, Dulaglutide, Suicidal ideation, Psychopathology, Depression, Anxiety, Cognitive dysfunction, Psychopharmacology, Bradford-Hill evaluation

## Abstract

**Purpose of Review:**

This review evaluates evidence linking both GLP-1 and GIP/GLP-1RAs’ exposure to a range of neuropsychiatric outcomes using the Bradford Hill criteria for causal inference.

**Recent findings:**

Glucagon-like peptide-1 (GLP-1) and the dual glucose-dependent insulinotropic polypeptide-GIP/GLP-1 receptor agonists (RAs) are incretin-based medications widely prescribed for type 2 diabetes mellitus and obesity, with demonstrated cardiometabolic benefit and rapidly expanding population exposure. In parallel, post-marketing surveillance and emerging translational research have raised questions regarding potential central nervous system effects, including neuropsychiatric adverse events and psychopharmacological properties. Integrating data from receptor pharmacology, preclinical neuroscience, randomized controlled trials, and observational and pharmacovigilance studies, the strength, consistency, biological plausibility, and experimental support for reported associations with depression, anxiety, suicidality, reward-related behaviour, cognitive effects and retinal/ocular related disturbances were here assessed.

**Summary:**

Implications for clinical risk–benefit assessment were discussed as well. Current evidence supports potential biological plausibility of a relationship between GLP-1 RAs and a range of psychopathological disorders but remains insufficient to establish causality for most neuropsychiatric outcomes, suggesting the need for prospective, mechanism-informed clinical studies.

## Introduction

Glucagon-like peptide-1 receptor agonists (GLP-1 RAs) have transformed the management of type 2 diabetes mellitus (T2DM) and obesity, demonstrating excellent levels of glycaemic control, weight reduction, and cardiovascular risk mitigation [16]. Agents such as liraglutide, semaglutide, dulaglutide, exenatide, lixisenatide, and albiglutide exert their primary effects via activation of the GLP-1 receptor which is a G protein–coupled receptor expressed in pancreatic, gastrointestinal, cardiovascular, and central nervous system (CNS) tissues [29]. Clinically, GLP-1 RAs constitute a class of synthetic analogues of the endogenous incretin hormone GLP-1, which is derived from pro-glucagon and secreted by enteroendocrine L-cells in response to nutrient ingestion (for an overview, see [83]). Furthermore, multi-agonist incretin-based therapies, exemplified by the dual glucose-dependent insulinotropic polypeptide-GIP/GLP-1 receptor agonist tirzepatide and the investigational triple (e.g., GIP/GLP-1/glucagon) agonist retatrutide, produce even more important metabolic and body-weight effects [3].

### GLP-1 and Dual GIP/GLP-1 RAs and Reward Pathway Modulation; a Psychopharmacological Perspective

Although GLP-1 RAs were not developed as centrally acting drugs, their documented [74] penetration of a range of brain regions and engagement of neural circuits regulating appetite and reward raise important questions regarding broader psychopharmacological effects. Indeed, despite diverse structures and actions, natural (e.g. food, sex) and pharmacological (e.g., nicotine, heroin, THC) rewards converge on the stimulation of the meso-cortico-limbic DA pathway involving the ventral tegmental area (VTA), nucleus accumbens (NAc), and prefrontal cortex (PFC). These circuits mediate reward salience, motivation, and reinforcement learning, and their alteration underlies compulsive drug seeking and loss of control [17, 50]. Overall, GLP-1 and GIP receptors are expressed in brain regions which are key nodes of the appetite and reward circuitries, including the VTA, NAc, lateral hypothalamus, and nucleus tractus solitarius, enabling both direct and polysynaptic influence on DAergic signaling [77].

Preclinical and human imaging studies of GLP-1RAs show modulation of DAergic and hedonic responses to food cues, suggesting direct or indirect central effects on reward pathways that may be associated with reduced food-seeking and altered motivational states (for an overview, see [7]. These central actions are likely partly responsible for appetite suppression and for changes in reward-driven behaviour.

Peripheral administration of GLP-1 RAs activates brainstem GLP-1–responsive neurons, which project to the VTA either directly or via hypothalamic relays. Within the VTA, GLP-1 receptor activation modulates DAergic neuron firing through cyclic adenosine monophosphate (cAMP)-dependent signalling and altered integration of glutamatergic and gamma-aminobutyric acid (GABA)-ergic inputs [61]. This results in attenuation of phasic DA release to the NAc in response to reward-predictive cues [32]. At the behavioural level, reduced DAergic signaling in the NAc core and shell translates into diminished incentive salience, reduced cue-triggered approach behaviour, and decreased compulsive reward seeking [38].

Preclinical research shows that activating GLP-1 receptors in the VTA and NAc specifically reduces cue-triggered spikes in DA activity, whilst leaving baseline (tonic) DA levels largely unaffected [32]. Pharmacologically, this pattern is consistent with modulation of *reward prediction error* (e.g., the difference between the reward actually received and the reward expected to receive) *signalling* rather than global DAergic suppression, distinguishing GLP-1 RAs from DA antagonists or monoaminergic antidepressants [8, 89]. Overall, GLP-1 RAs, and potentially dual GIP/GLP-1 agonists, should be conceptualized as neuromodulators that recalibrate reward sensitivity through indirect DAergic control rather than as agents that directly induce mood elevation or suppression [77].

Human functional Magnetic Resonance Imaging (MRI) studies confirmed that GLP-1 RA administration may be associated with reduced activation of reward-related regions, including the NAc, orbitofrontal cortex, and insula, in response to food cues, particularly in the postprandial state [81]. Positron emission tomography (PET) imaging studies further suggest that these effects occur without significant displacement of DA D2/3 receptor ligands, supporting an indirect modulatory mechanism rather than direct DAergic receptor engagement. Collectively, these findings align with a model in which GLP-1 RAs dampen reward responsivity through upstream control of DA neuron firing and network-level salience attribution [32]. Moreover, the potential GLP-1RA’s influence on serotonin (5-HT) circuitry should not be neglected; this could influence both mental well-being and metabolic health via direct or indirect pathways, for e.g. through effects on gut-brain axis, hippocampus and pro-opiomelanocortin (POMC) circuitry [18, 48].

Dual GIP/GLP-1 receptor agonists introduce additional complexity to reward pathway modulation. GIP receptors are expressed in overlapping but distinct CNS regions and may exert permissive or counter-regulatory effects on reward signalling. Preclinical data suggest that combined GIP and GLP-1 receptor activation can produce more pronounced reductions in reward-driven feeding than GLP-1 receptor activation alone, potentially reflecting synergistic effects on hypothalamic and mesolimbic circuits [30]. From a pharmacological perspective, this raises the possibility that dual agonists may exert quantitatively stronger or qualitatively distinct effects on motivation and reward, with uncertain implications for neuropsychiatric tolerability. Importantly, whether these differences translate into clinically meaningful divergence in mood, motivation, or addictive behaviour in humans remains insufficiently characterized.

### Neuropsychiatric Adverse Events Associated with GLP-1 and Dual GIP/GLP-1 RAs

Overall, neuropsychiatric adverse events associated with both GLP-1 and dual GIP/GLP-1 RAs have attracted increasing levels of attention, particularly as these agents are widely used in diabetes and obesity management and are being explored for neuropsychiatric indications [19].

#### Mood and Affective Symptoms

In general, the desire to achieve an ideal body image is prevalent in society, and individuals with obesity or diabetes may face additional challenges to this respect. Anti-obesity medications, with their weight-reducing effects, can lead to body image enhancement and improved self-esteem, positively impacting mental well-being for many individuals [23].

Post-marketing surveillance and pharmacovigilance databases have, however, reported cases of depressive symptoms, anxiety, and, rarely, suicidal ideation in patients receiving GLP-1 RAs. Indeed, Guirguis et al., [36] showed a potential increased association between liraglutide, semaglutide, and tirzepatide and suicidal thoughts/actions. Ruggiero et al., [70] showed similar findings and uncovered a notable prevalence of suicidal events linked to semaglutide and liraglutide, with markedly higher reporting probabilities compared to other GLP-1 RAs. Their research suggested as well a potential correlation between the risk of suicidal events and the utilization of higher doses of GLP-1 RA for weight management [70]. Similarly, in the research conducted by McIntyre et al., (2024), an uneven reporting pattern emerged regarding suicidal ideation and depression/suicidal events associated with both semaglutide and liraglutide in comparison to metformin and insulin.

There is however a well-established correlation between T2DM/obesity and depressive disorders [62]; those factors associated with these conditions may have contributed to the observed, pharmacovigilance-related, instances of suicidality thoughts and actions.

To better assess the above findings, Chen et al., [12] conducted a subgroup analysis specific to each of six GLP-1 RAs, i.e., liraglutide, lixisenatide, exenatide, albiglutide, semaglutide and dulaglutide; tirzepatide was not included. Although some 199; 134; and 106 suicide/self-injury reports were found to be respectively associated with liraglutide; exenatide; and semaglutide, they found that the overall class of GLP-1 RAs, per se, did not demonstrate an increased risk of suicidal/self-injurious events. Furthermore, Wang et al., [84] conducted a retrospective cohort study in the United States utilizing electronic health records (EHR) extracted from an Analytics Network. Their study revealed a lower risk for both occurrence and recurrence of suicidal ideation in patients prescribed semaglutide when compared to those using non-GLP1 RA medications for either obesity or diabetes, which also included metformin, hence confirming previous suggestions [36].

GLP-1 receptors are expressed in limbic and brainstem regions implicated in mood regulation, and modulation of DAergic and stress-related pathways could theoretically influence affective states. However, it is essential to interpret possible medication-associated mood alterations within the broader context [64] of anti-obesity medications’ use. In fact, several of these molecules, such as selective serotonin reuptake inhibitors (SSRIs) and the naltrexone/bupropion combination have been associated with potential psychological side-effects, including changes in mood and the occurrence of suicidal ideation [76].

Further hypotheses have been proposed to explain the putative, potential, link between GLP-1 RAs and mental health issues [7].

One of the psychological conditions in those considering taking GLP-1 RAs, particularly semaglutide [13], is body dysmorphic disorder (BDD), e.g., a condition characterized by obsessive thoughts and preoccupations with perceived flaws or defects in one’s appearance; psychological distress, anxiety, depression [82] and increased risk of suicidal ideation and behaviour [15].

Using a pharmacovigilance approach, which in itself may present with some limitations (e.g., level of reporting in association with how long the medicine has been on the market; popularity of the molecule on social media and its licensing, or not, for the management of obesity), our research group identified both a potential for medication misuse and a development of clinical rebound/withdrawal symptoms with semaglutide [13]. Hence, one would assume that those affected by BDD and/or emotional eating, as well as misusing with a GLP-1 RA, would be at higher risk of depression and suicide (e.g., cohort bias).

Furthermore, rapid weight loss or adjustment to a new body image may trigger both biological and psychological responses that could influence suicidal ideation. Indeed, it is well-known that serum lipid levels, and specifically low levels of cholesterol, could have an impact on the development of both suicidality [22] and impulsivity [78]. Indeed, decreased cholesterol levels may have a negative impact on serotonergic activity, particularly by inducing alterations in the structure and function of cell membranes within the CNS [22].

Recently, however, the Food and Drug Administration (FDA) conducted a comprehensive meta-analysis of clinical trials that compared GLP-1 RA drugs with placebo to assess the risk of suicidal ideation/behaviour (SI/B). The analysis included 91 trials enrolling 107,910 patients, 60,338 receiving a GLP-1 RA and 47,572 receiving placebo. Findings showed no increased risk for SI/B with GLP-1 RA use compared with placebo. Similar results were seen for other psychiatric adverse events including anxiety, depression, irritability, and psychosis (US FDA, 2026). The risk of intentional self-harm with GLP-1 RAs was also investigated in a retrospective cohort study using administrative health care claims data from the FDA Sentinel System. The study population included 1,161,983 individuals initiated on a GLP-1 RA and 1,081,155 started on a sodium-glucose cotransporter 2 inhibitor (SGLT2i). Results showed no increased risk of intentional self-harm among GLP-1 RA users when compared with SGLT2i users. In addition to these studies, the FDA examined observational and pooled studies evaluating the relationship between GLP-1 RAs and SI/B. Taken all together, in line with recent evidence [65] the Agency concluded there may be no causal relationship between these medications and the occurrence of this adverse event.

One could still argue that if, at present, evidence does not support a clear, class-wide, pro-depressogenic effect, vigilance may still be warranted in vulnerable patients, and especially so in subjects with pre-existing mood disorders. In fact, focusing on a large amount of trial-related data may obscure the occurrence of single clinical episodes, putatively being related to the index individual’s psychobiological vulnerability. Furthermore, for some subjects, some nutrients (e.g., chocolate) may indeed constitute a ‘comfort item’ being used to cope with depressive feelings [72]. When being prescribed with GLP-1/GIP RAs, due to the food-related blunted dopamine (DA) reward response, food ingestion will not be a source of satisfaction any longer for these vulnerable subjects and hence the putative, theoretical, risk of depression development in some of them. Indeed, Sa et al., [71] recently carried out a retrospective chart review of adults prescribed with GLP-1 RAs. Among 226 patients (mean age 53.5 years; 66.8% female; mean BMI 33.6 kg/m^2^), semaglutide was most frequently prescribed (73%). Although most remained stable (depression: 73.6%; anxiety: 78.5%), a new-onset depressive episode occurred in 9.0% and a generalized anxiety disorder in 8.7% of affected patients, with mean latencies of 15.8 and 16.8 months, respectively. Adjustment disorder, Attention Deficit and Hyperactivity Disorder (ADHD), and insomnia also emerged in a subset, with ADHD showing the shortest latency to onset (7.8 months). In line with this, Sharafshah et al., [75] suggested that GLP-1 RAs may represent a double-edged sword, potentially triggering anti-consumption effects but also exacerbating depressive phenotypes in some individuals.

For tirzepatide, however, emergent observational analyses have not shown a consistent increase in suicide risk and one recent real-world study [88] reported a lower observed risk of suicidal ideation or attempts compared with other anti-obesity medications.

#### Anxiety, Agitation, and Sleep Disturbances

With GLP-1 RAs, reports of anxiety, restlessness, and insomnia are uncommon but have been described [85]. One could argue that these symptoms may be secondary to gastrointestinal adverse effects, weight loss–related metabolic changes, or central GLP-1 receptor activation. In clinical trials, rates of anxiety-related adverse events are generally low and comparable to placebo. No consistent signal for psychosis or mania has emerged [85].

Ouyang et al., [65] carried out a two-sample Mendelian randomization, Linkage Disequilibrium Score Regression (LDSC) and Bayesian co-localization to assess causal associations between GLP-1RAs and a range of mental disorders. GLP-1RAs resulted in not being causally related to anxiety (OR = 0.870, P_FDR_ = 0.250), or bipolar affective disorders (OR = 0.931, P_FDR_ = 1.00). The meta-analysis revealed no significant difference between the GLP-1 RAs exposure group and the control group in terms of anxiety (OR = 1.03, P_OR_ = 0.93). Conversely, Alibilli et al., [4] quantitatively analyzed temporal and co-occurrent GLP-1 RA adverse event trends through discussions of GLP-1 RA weight loss medications on Facebook from 2022 to 2024. They found that anxiety, depression, and insomnia were frequently mentioned and were highly correlated with each other (50–100 co-occurrence mentions). Similarly, Arillotta et al., [7] assessed the possible impact of GLP-1 RAs on mental health as being perceived and discussed in popular open platforms, e.g., Reddit, YouTube and TikTok. Most represented comments related to insomnia (*n* = 620 matches); anxiety (*n* = 353); depression (*n* = 204); and mental health issues in general (*n* = 165). After the initiation of GLP-1 RAs, losing weight was associated with either a marked improvement or, in some cases, a deterioration, in mood and an increase/decrease in anxiety/insomnia.

#### Cognitive and CNS Effects

With GLP-1 and GIP/GLP-1 RAs medications, headache and dizziness are relatively common, whilst being typically mild and transient [85]. There is no robust evidence of cognitive impairment associated with GLP-1 RAs; conversely, preclinical and early clinical studies suggest potential neuroprotective or pro-cognitive effects, particularly in neurodegenerative disorders [40]. Thus, current data does not indicate clinically meaningful cognitive toxicity.

In line with this, Lee et al., [51] carried out a systematic review focusing on the treatment effects of GLP-1 RAs in various neurocognitive and psychiatric disorders; some 22 studies were identified, with a total of 186,847 participants included. GLP-1RAs were found to be associated with a meaningful improvement of both cognitive and affective functioning (e.g., memory), which, in some cases, was sustained beyond exposure to the agent.

It is a reason of concern, however, that recent pharmacovigilance research has highlighted a possible onset of neurological, eye-related, disorders in association with both GLP-1 and dual GIP/GLP-1 receptor agonists. Indeed, Murray et al., [63] carried out a retrospective clinical cohort disproportionality analysis of eye-related disorders’ reports made in recent years to the Food and Drug Administration Adverse Event Reporting System (FAERS), in cases with and without T2DM. Reporting Odds Ratios (RORs) > 4.000 and 95% confidence intervals were calculated, with metformin and orlistat as controls. In non-T2DM patients, in comparison with metformin, semaglutide showed increased reporting of retinopathy (ROR: 9.424 [1.081–3.406]) and retinal hemorrhage (ROR: 10.253 [0.319–4.336]); tirzepatide showed increased reporting of eye swelling (ROR: 6.475 [0.407–3.329]); and liraglutide showed increased reporting of cataract (ROR: 9.628 [1.387–3.142]) and macular degeneration (ROR: 9.557 [0.110–4.405]).

### Effects on Food/Liquid intake

Given their central effects on appetite and reward circuitry, GLP-1 RAs may alter food-related reward processing. In most cases, this manifests as reduced appetite and decreased hedonic eating. Whilst being generally therapeutic, abrupt changes in reward sensitivity could theoretically influence mood or motivation in susceptible individuals [81].

Indeed, Ouyang et al., [65] recently carried out a meta-analysis which showed a significant difference in eating disorders (ED) (SMD = -0.71, P_SMD_ = 0.006) between the GLP-1 RAs exposure and the control group. Conversely, Maynard et al., [58] carried out a retrospective observational cohort study which included adults with obesity and found that GLP1 RAs did not significantly worsen ED risk or psychological distress.

An issue of interest here is *emotional eating*, which is the tendency to use food as a coping mechanism for emotional distress. Indeed, psychological factors like unhealthy coping mechanisms may play a role in the development and maintenance of obesity whilst also possibly impacting on mental health [62].

However, with food reward blunting being associated with the intake of GLP-1/GIP RAs, one could argue that such coping mechanism may be disrupted. A similar issue occurred with rimonabant, which was designed to reduce appetite and food intake by blocking the CB1 receptors in the brain, which are involved in the regulation of appetite and energy balance. However, rimonabant was withdrawn from the market due to safety concerns related to increased risk of depression, suicidal ideations [69], and completed suicide [9]. The drug’s antagonistic action on the central endocannabinoid system may possibly impact on DA, blunting the food-associated activation of the reward pathways [42], resulting in anhedonia, depression and suicidal feelings.

Primary polydipsia, a drinking behavior characterized by excessive liquid intake which is related to dysfunctions of reward and compulsivity and which is typically identified in people with mental and/or neurodevelopmental disorders [44], has shown symptoms’ improvement in a controlled trial involving dulaglutide [86].

#### Substance Use

Recent evidence suggests that GLP-1 therapies may be beneficial for treating alcohol and other substance use disorders (SUDs). GLP-1 and GIP RAs modulate DAergic signalling within the mesolimbic reward circuitry and may attenuate the reinforcing properties of addictive substances, thereby representing a potential therapeutic strategy for SUDs [47, 67]. More specifically, activation of GLP-1 receptors appears to counteract the mesolimbic DA dysregulation (for a thorough overview, see [83]), a core neurobiological mechanism underlying addiction and relapse vulnerability [37]. In alcohol use disorder (AUD), GLP-1 RAs such as semaglutide and liraglutide reduce alcohol-induced DA release in the NAc and attenuate relapse-like drinking in rodents ([6] b). Similar effects have been observed with other substances. Liraglutide reduces nicotine-seeking behaviour, while exenatide decreases heroin-induced reinstatement in preclinical models, suggesting that GLP-1–mediated modulation of reward processing extends beyond alcohol [37]. In contrast, evidence regarding cocaine use disorder remains less consistent, with some studies indicating that therapeutic efficacy may depend on substance-specific neurobiological mechanisms within the mesolimbic system, particularly differences in DAergic signaling dynamics (Hernandez et al., 2019; Schmidt et al., 2016).

The mechanisms underlying differential efficacy across substances are incompletely understood. Factors such as sex, baseline severity of substance use, and comorbid metabolic conditions may influence treatment response, yet their precise contributions require clarification [80]. Although short-term trials suggest acceptable tolerability, the durability of therapeutic effects in long-standing addiction remains uncertain [80]. Additionally, the optimal integration of GLP-1 RAs into existing treatment frameworks, either as monotherapy or adjunctive therapy, has yet to be systematically evaluated. Preclinical data indicate potential synergistic effects when combined with agents targeting complementary pathways, such as ghrelin antagonists [1]; however, these approaches require rigorous validation in adequately powered clinical trials.

Very recently, Cai et al., [11] investigated whether starting GLP-1 RAs is associated with lower risks of developing SUDs and improved outcomes in people who already have SUDs. Seven trials examined the incidence of different SUDs among individuals without a prior history (protocol 1), and one trial assessed adverse outcomes among individuals with pre-existing SUDs (protocol 2). From a base population of 606,434 veterans with T2DM, participants were followed for up to three years. Protocol 1 included 524,817 individuals initiating either GLP-1 RAs or sodium–glucose cotransporter-2 (SGLT-2) inhibitors. Protocol 2 included participants with existing SUDs who initiated GLP-1 RAs (16,768) or SGLT-2 inhibitors (64,849). Compared with SGLT-2 inhibitors, GLP-1 RAs were associated with lower risks of incident alcohol, cannabis, cocaine, nicotine, opioid, and other SUDs, as well as a reduced overall risk of developing any SUD. Among individuals with pre-existing SUDs, GLP-1 RA initiation was also linked to lower risks of SUD-related emergency department visits, hospital admissions, mortality, drug overdose, and suicidal ideation or attempts. A range of GLP-1 dual GIP/GLP-1 RA-related randomized clinical trials (RCTs) are ongoing or about to start [25]. 

Neuropsychiatric adverse events associated with GLP-1 and dual GIP/GLP-1 RAs are summarized in Table 1. 

**Table 1:** Neuropsychiatric adverse events associated with GLP-1 and dual GIP/GLP-1 RAs

	Adverse events / effects	Evidence summary	Key considerations / mechanisms
Mood and affective symptoms	Depression, anxiety, suicidal ideation/behaviour (rare), mood changes	Pharmacovigilance signals suggest a possible association (liraglutide, semaglutide, tirzepatide); some studies show increased reporting of suicidality, especially at higher doses. However, large meta-analyses and FDA evaluations show no causal association and, in some cases, reduced risk vs. comparators.	Confounding by underlying obesity/T2DM (linked to depression); GLP-1 receptor activity in mood-related brain regions; rapid weight loss, cholesterol reduction, and serotonergic effects; vulnerability in predisposed individuals (e.g., prior psychiatric illness, body dysmorphic disorder).
Anxiety, agitation, sleep disturbances	Anxiety, restlessness, insomnia (uncommon)	Clinical trials show low incidence, similar to placebo; no consistent signal for psychosis or mania. Social media and observational data report frequent co-occurrence of anxiety, depression, and insomnia. Mendelian randomization studies show no causal link.	May be secondary to GI effects, metabolic changes, or central receptor activation; subjective reporting variability; bidirectional relationship with weight loss outcomes.
Cognitive and CNS effects	Headache, dizziness (common, mild); no cognitive impairment; possible cognitive improvement	No robust evidence of cognitive toxicity; some studies suggest neuroprotective and pro-cognitive effects (e.g., improved memory).	Central GLP-1 activity may support neuroprotection; transient CNS symptoms likely non-specific.
Neurological / sensory (visual) signals	Retinopathy, retinal haemorrhage, eye swelling, cataract, macular degeneration (signals)	Pharmacovigilance analyses report disproportionate reporting of eye-related disorders with semaglutide, tirzepatide, and liraglutide (including non-T2DM populations).	Mechanism unclear; requires further validation; may relate to metabolic or vascular changes.
Eating behaviour & reward	Reduced appetite, decreased hedonic eating; possible impact on emotional eating; theoretical risk of anhedonia	Generally therapeutic (weight loss); mixed evidence on eating disorders; some meta-analyses show improvement; others show no worsening of risk.	Modulation of reward circuitry and DA signalling; disruption of food as coping mechanism may affect vulnerable individuals; parallels drawn with prior anti-obesity drugs (e.g., rimonabant).
Substance misuse / addiction	Potential reduction in alcohol, nicotine, opioid, and other substance use; reduction in relapse levels and SUD-related harms	Preclinical and clinical data suggest beneficial effects on substance use disorders; large cohort studies show reduced incidence of SUDs and improved outcomes in affected individuals.	Modulation of mesolimbic DA pathways; attenuation of reward from addictive substances; possible role as adjunct therapy; possible significant levels of variability by substance type and patient factors.

*CNS* Central Nervous System, *FDA* Food and Drug Administration, *GI* Gastrointestinal *GLP* Glucagon-like Peptide, *SUD* Substance Use Disorder, *T2DM* Type 2 Diabetes Mellitus

### Aims

We aimed here at applying the Bradford Hill (BH) criteria [39] to assess whether observed associations between GLP-1; GIP/GLP-1 RAs; and neuropsychiatric outcomes (namely: depression; suicidal ideation; anxiety; cognitive and CNS effects; eating disorders; substance misuse) plausibly reflect causal drug effects or are better explained by confounding, bias, or disease-related factors.

## Methods

This review synthesized evidence from: molecular and systems-level studies of both GLP-1 and GIP receptor signalling in the CNS; animal models assessing behavioral and affective outcomes; randomized controlled trials reporting neuropsychiatric adverse events; observational cohort studies; case–control analyses; and post-marketing pharmacovigilance data. Neuropsychiatric outcomes of interest included: depression; suicidal ideation; anxiety; cognitive and CNS effects; eating disorders; substance misuse.

Medline/PubMed, Embase, Web of Science, Scopus, and PsycINFO, alongside Google Scholar were searched for studies using the terms “GLP-1 receptor agonists; “GLP-1 RAs”; “dual GIP/GLP-1 RAs”; “triple GIP/GLP-1/glucagon RAs”; “semaglutide”; “tirzepatide”; “liraglutide”; “exenatide”; “retatrutide”; “dulaglutide”; “suicidal ideation”; “anxiety”; “depression”; “addiction”; “reward pathways”; “psychopathology”; and “psychopharmacology”. No formal meta-analysis was conducted due to heterogeneity of outcome definitions and study designs.

### Bradford Hill criteria

The BH criteria are a set of principles used in epidemiology to help judge whether an observed association is likely to be causal and not just a correlation [39]. The criteria include: *Consistency*,* Specificity*,* Temporality*,* Biological Gradient*,* Biological Plausibility*,* Coherence*,* Experimental Evidence*,* and Analogy.* Multiple criteria reinforce the same causal pathway. Although temporality is essential (e.g., without it, causation is impossible), satisfaction of all 9 principles/criteria is not needed to infer causality. These criteria are especially useful when randomized trials are unethical or impossible. Overall, the BH evaluation can be conceived as a structured way of assessing whether an observed association is likely to be causal. Although the methodology does not refer, per se, to a strict protocol, it consists in a systematic appraisal across the nine BH criteria. Hence, one would argue that BH evaluations are narrative and interpretative.

## Results

Across large cardiovascular outcome trials and dedicated obesity trials, absolute rates of reported neuropsychiatric adverse events associated with GLP-1 RAs and dual GIP/GLP-1 RAs are low, and relative risks compared with placebo are generally modest. Indeed, in major trials such as LEADER, SUSTAIN-6, REWIND, and SELECT, which collectively include many thousands of participants with long-term follow-up, psychiatric adverse events, including depression, anxiety, and suicidality, were infrequently reported and did not emerge as leading causes of treatment discontinuation [33, 52, 56, 57]. Similarly, in large obesity trials such as STEP and SURMOUNT, rates of mood-related adverse events were low and broadly comparable between active treatment and placebo groups, despite substantial weight loss and prolonged exposure to centrally active incretin-based therapies [45, 85]. Post-marketing pharmacovigilance analyses and spontaneous adverse event reporting systems have however identified signals related to depression and suicidal ideation for GLP-1 RAs and dual GIP/GLP-1 RAs [36]. Nonetheless, these signals are characterized by small effect sizes, low absolute event counts, and substantial susceptibility to reporting bias, and differential surveillance in populations with obesity or diabetes, which are conditions themselves associated with elevated baseline psychiatric risk [21, 28]). Taken together, evidence from randomized trials and real-world pharmacovigilance suggests that while psychiatric adverse events warrant attention, they do not appear to represent large or common safety signals at the population level for GLP-1–based or dual incretin therapies. Overall, the above observations may limit support for strong associations.

### Consistency

Preclinical studies consistently demonstrate that central GLP-1 signalling exerts robust effects on stress- and reward-related neural circuits. In rodent models, GLP-1 RA activation within meso-cortico-limbic regions such as the ventral tegmental area, Nac, lateral septum, and hypothalamus modulates DAergic transmission, attenuates reward-driven behaviour, and alters motivational salience, particularly in the context of food and substance reward [2, 5, 55]. Parallel work demonstrates that GLP-1 RAs’ signaling interacts with stress-responsive systems, including the hypothalamic–pituitary–adrenal (HPA) axis, influencing corticotropin-releasing hormone activity, glucocorticoid secretion, and behavioural responses to acute and chronic stress [41, 87]. These preclinical findings are further supported by evidence that GLP-1 RAs reduce neuroinflammation and enhance synaptic plasticity in limbic and hippocampal circuits, mechanisms that may indirectly influence affective and stress-related phenotypes [43, 53, 59].

In contrast, translation of these findings to humans has yielded heterogeneous and sometimes conflicting results. Clinical trials and observational studies of GLP-1 RAs report neutral, beneficial, or adverse psychiatric outcomes, with variability across measures of mood, anxiety, anhedonia, and suicidality [19, 60]. Some studies suggest improvements in depressive symptoms, cognitive function, or quality of life, potentially mediated by weight loss, improved glycaemic control, or reduced systemic inflammation, whereas others report no significant psychiatric effects or raise concerns about mood changes in specific subgroups ([60]; Yabe and Seino, 2021). Importantly, inconsistencies across human studies likely reflect differences in study design, duration of exposure, psychiatric assessment tools, baseline mental health status, and whether central GLP-1 RAs’ engagement occurs at clinically used doses. Together, these observations underscore a clear translational gap between robust preclinical evidence for GLP-1–mediated modulation of stress and reward circuitry and the variable psychiatric outcomes observed in human populations.

### Specificity

Reported psychiatric outcomes associated with GLP-1 RAs are largely non-specific and overlap substantially with the elevated baseline psychiatric risk observed in populations with obesity and T2DM. Depression, anxiety, sleep disturbance, and suicidal ideation, events most commonly cited in clinical trials and pharmacovigilance databases, are highly prevalent in these populations independent of pharmacological treatment, driven by complex interactions between metabolic dysfunction, chronic inflammation, psychosocial stressors, stigma, and comorbid medical illness [34]. Large randomized cardiovascular outcome trials and obesity trials have not demonstrated a consistent or syndrome-specific psychiatric signal attributable to GLP-1 RAs, with reported events occurring at low absolute frequencies and without clear dose–response relationships or temporal clustering suggestive of causality [52, 56, 57, 85].

Importantly, spontaneous adverse event reports related to mood or suicidality lack phenotypic specificity and are vulnerable to confounding by indication, differential surveillance, and notoriety bias, particularly in patient groups already at increased psychiatric risk and subject to heightened clinical monitoring during weight loss interventions [21, 28]. Observational studies further suggest that changes in mood following GLP-1 RAs’ initiation may reflect indirect effects of weight loss, caloric restriction, glycaemic improvement, or changes in concomitant medications rather than direct neuropsychiatric toxicity [60]. Taken together, the non-specific nature of reported psychiatric outcomes and their substantial overlap with background risk profiles may confirm the difficulty of disentangling drug-related effects from underlying vulnerability in populations treated with GLP-1 RAs. Overall, specificity of an impact on a single diagnostic entity is lacking.

### Temporality

Case reports and a limited number of observational studies have described the temporal emergence of psychiatric symptoms following initiation of GLP-1 RAs, with some reports noting partial or complete symptom resolution after treatment discontinuation. Reported symptoms include depressed mood, anxiety, insomnia, emotional blunting, and suicidal ideation, typically occurring within weeks to months of treatment initiation [21, 60]. In several individual case narratives, dechallenge–rechallenge patterns have been described, wherein symptoms improved upon cessation of the GLP-1 RA and recurred after re-exposure, raising the possibility of a drug-related effect in susceptible individuals [21]. However, such reports are inherently limited by small sample sizes, lack of standardized psychiatric assessment, and incomplete characterization of baseline mental health status, concomitant medications, and psychosocial stressors.

Observational cohort studies and analyses of spontaneous adverse event reporting systems have similarly identified temporal associations between GLP-1 RA exposure and reported psychiatric events, but these findings remain inconsistent and are vulnerable to confounding, particularly in populations with obesity or diabetes who carry a high baseline risk of mood disorders and are subject to intensified clinical monitoring during weight loss or metabolic interventions [28, 34].

Overall, these observations may support, but not prove, temporality.

### Biological Gradient

Evidence supporting clear dose–response relationships between GLP-1 RA exposure and psychiatric outcomes remains limited. Across randomized cardiovascular outcome trials and obesity trials, psychiatric adverse events have been reported at low absolute frequencies and without consistent increases at higher doses or longer durations of treatment, limiting inference regarding dose dependence [52, 56, 85]. Observational studies and pharmacovigilance analyses have similarly failed to demonstrate robust dose–response gradients, in part due to imprecise exposure measurement, heterogeneous dosing regimens, and reliance on spontaneous reporting systems that lack denominator data and standardized dose stratification [21, 28]).

Nonetheless, pharmacokinetic and pharmacodynamic differences among GLP-1 RAs, including molecular size, lipophilicity, half-life, and blood–brain barrier permeability, suggest that agents may differ meaningfully in CNS exposure and receptor engagement, potentially giving rise to gradients of central effects that have not been systematically tested in clinical populations [14, 19]. Longer-acting agents and higher-potency formulations used in obesity treatment may achieve sustained plasma concentrations and prolonged GLP-1 receptor activation, which may increase the likelihood of central receptor engagement compared with shorter-acting agents, although direct comparative neuropsychiatric data are lacking [60]. Together, these observations highlight an important evidence gap, e.g., whilst biological plausibility exists for dose- and exposure-related central effects of GLP-1 RAs, current clinical and post-marketing data are insufficient to define reliable dose–response relationships for psychiatric outcomes.

### Biological Plausibility

Pharmacological activation of central GLP-1 RAs attenuates mesolimbic DA release in response to rewarding stimuli without suppressing basal DA tone, thereby reshaping reward processing and motivational behaviour [5, 55]. In parallel, GLP-1 RAs signalling influences serotonergic and other monoaminergic systems involved in affective and cognitive regulation [41, 55]. Beyond neurotransmitter modulation, GLP-1R activation interacts with the HPA axis; indeed, central GLP-1 signalling can regulate corticotropin-releasing hormone activity and glucocorticoid secretion, implicating GLP-1 RA pathways in stress responsiveness and neuroendocrine homeostasis [41, 87]. GLP-1 RA activation also exerts pronounced anti-inflammatory effects within the CNS. GLP-1 RA agonists suppress microglial activation and reduce the production of pro-inflammatory cytokines such as TNF-α and IL-1β, contributing to a neuroprotective environment in models of neurodegeneration and metabolic-inflammatory brain dysfunction [43, 53].

At the synaptic level, GLP-1 RA signaling enhances synaptic plasticity by promoting long-term potentiation, facilitating AMPA receptor trafficking, and activating intracellular cascades involving cAMP, protein kinase A, and cyclic adenosine monophosphate response element-binding protein (CREB) phosphorylation [20]. These effects support synaptic integrity and cognitive function, particularly in hippocampal circuits vulnerable to aging and neurodegenerative pathology [53, 59]. Collectively, these findings position GLP-1 RAs’ activation as a multifaceted neuro-modulatory mechanism linking metabolic signals to reward processing, stress regulation, neuroinflammatory control, and synaptic plasticity [19, 55]. Overall, these mechanisms plausibly link GLP-1 RAs to mood and behavioural effects.

### Coherence

Proposed neuropsychiatric effects of GLP-1 RAs are broadly coherent with current understanding of gut–brain signaling and the well-established bidirectional relationship between metabolic and psychiatric disorders. GLP-1 is a key component of the gut–brain axis, acting through vagal afferents, brainstem nuclei, and direct central receptor engagement to influence appetite, reward processing, stress responsivity, and affective regulation [19, 55]. These pathways overlap substantially with neural circuits implicated in mood and anxiety disorders, including meso-cortico-limbic DA systems, hypothalamic stress circuitry, and limbic structures involved in emotional salience [2, 41]. In parallel, obesity and T2DM are strongly associated with depression and anxiety through shared mechanisms such as chronic low-grade inflammation, hypothalamic–pituitary–adrenal axis dysregulation, insulin resistance, and altered monoaminergic signaling, providing a biological substrate through which metabolic interventions may influence psychiatric outcomes [34].

Within this framework, both beneficial and adverse neuropsychiatric effects of GLP-1 RAs are biologically plausible. Improvements in mood or cognition may arise indirectly via weight loss, improved glycaemic control, reduced systemic and neuroinflammation, and enhanced synaptic plasticity, whereas adverse symptoms in susceptible individuals could reflect perturbations of central reward or stress circuits during rapid metabolic change [43, 60]. Thus, reported neuropsychiatric phenomena associated with GLP-1 RA therapy are consistent with current models of metabolic–psychiatric comorbidity and gut–brain communication, supporting mechanistic plausibility even as causal attribution at the population level remains uncertain.

### Experimental Evidence

Few trials prospectively evaluated psychiatric outcomes. Improvements in quality of life and depressive symptoms observed during treatment with GLP-1 RAs are often observed alongside weight loss and associated metabolic improvements, rather than being attributed solely to direct central neuropsychiatric drug effects. Substantial evidence indicates that intentional weight reduction in individuals with obesity or T2DM is independently associated with improvements in depressive symptoms, self-esteem, physical functioning, and health-related quality of life, irrespective of the specific intervention used to achieve weight loss [24, 27]. In large GLP-1 RA obesity trials, improvements in patient-reported outcomes often correlate more strongly with the magnitude of weight loss than with treatment per se, suggesting mediation through somatic, functional, and psychosocial pathways such as reduced pain, improved mobility, enhanced body image, and decreased obesity-related stigma [49, 85]. However, these observations do not exclude the possibility of additional direct treatment-related effects. Moreover, improvements in glycaemic control, sleep quality, cardiovascular risk factors, and systemic inflammation, all common consequences of GLP-1 RA–induced weight loss, are themselves associated with reduced depressive symptom burden and improved emotional well-being (Moulton et al., 2015; [34]). Importantly, clinical trials have not consistently demonstrated mood improvements in the absence of significant weight loss, nor have they identified clear dose–response relationships between GLP-1 RA exposure and depressive symptom change after adjusting for weight reduction, which is consistent with, but does not by itself prove, an indirect mechanism [52, 60]. Taken together, these findings suggest that reported benefits in mood and quality of life during GLP-1 RA therapy are more plausibly explained in many cases by downstream effects of weight loss and metabolic improvement rather than by primary central antidepressant actions.

#### Analogy

Peripherally administered hormones, cytokines, and metabolic mediators can influence mood, cognition, reward processing, and stress-related circuits, supporting a limited analogy for potential central neuropsychiatric effects of GLP-1 RAs. For example, interferon-α and interleukin-2, can induce depressive, anxiety, and cognitive symptoms through cytokine signaling across the blood–brain barrier, activation of microglia, and alterations in monoaminergic neurotransmission, while exogenous corticosteroid therapy is associated with depression, anxiety, mania, and psychosis through dysregulation of hypothalamic–pituitary–adrenal axis signaling and limbic circuitry [10, 66]. Likewise, insulin influences cognition and mood, and leptin modulates reward processing, motivation, and affective state, with leptin deficiency or resistance linked to depressive phenotypes [26, 54]. Taken together, these observations suggest biological plausibility by analogy, although the magnitude and clinical relevance of any GLP-1 RAs-related neuropsychiatric effects remain uncertain [19, 55]. 

An overview of the Bradford Hill apprach findings is provided in Table 2. 

**Table 2:** Neuropsychiatric adverse events associated with GLP-1 and dual GIP/GLP-1 RAs; an overview of the Bradford Hill approach findings

Bradford Hill Criterion	Neuropsychiatric Outcomes	Overall Interpretation
Strength of Association	Generally weak and inconsistent associations; some pharmacovigilance signals but large analyses do not confirm increased risk.	Partially met – strong consistency in animal models, but inconsistent translation to human outcomes limits causal inference. Weak–moderate levels of association.
Consistency	Strong and consistent preclinical evidence shows GLP-1 signalling modulates stress, reward, and DAergic pathways. However, human studies are heterogeneous, reporting beneficial, neutral, or adverse psychiatric outcomes depending on study design, population, and measures.	Partially met; inconsistency limits causality.
Specificity	Psychiatric outcomes (depression, anxiety, insomnia, suicidality) are non-specific and highly prevalent in populations with obesity and T2DM. No clear symptoms/signs uniquely attributable to GLP-1 RAs.	Not met; lack of specificity.
Temporality	Case reports and observational data show symptom onset after treatment initiation, sometimes with dechallenge–rechallenge patterns. However, evidence is limited and confounded.	Partially met.
Biological Gradient (Dose–response)	No consistent dose–response relationship; theoretical CNS exposure differences not clinically confirmed.	Not established – insufficient evidence.
Biological Plausibility	Strong mechanistic basis: GLP-1 RAs influence DAergic, serotonergic, HPA axis signalling, neuroinflammation, and synaptic plasticity in brain regions regulating mood and behaviour.	Strongly supported.
Coherence	Findings align with current understanding of the gut–brain axis and the link between metabolic disorders and psychiatric conditions. Both beneficial and adverse effects are biologically plausible.	Supported; aligns with existing biological frameworks.
Experimental Evidence	Few trials specifically assess psychiatric outcomes. Observed improvements in mood and quality of life are largely mediated by weight loss and metabolic improvements, not direct CNS effects.	Limited; moderate levels for support; indirect mechanisms predominate.
Analogy	Supported by other agents (e.g., cytokines, glucocorticoids, insulin, leptin) affecting mood and cognition.	Supported – analogy strengthens plausibility.

*CNS* Central Nervous System, *GLP-1* Glucagon-like Peptide-1, *GLP-1 RA* Glucagon-like Peptide-1 Receptor Agonist, *HPA* Hypothalamic-Pituitary-Adrenal, *SUD* Substance Use Disorder, *T2DM* Type 2 Diabetes Mellitus

## Discussion

Other reviews have examined neuropsychiatric outcomes, safety signals, and regulatory evidence related to GLP-1 RA; however, to the best of our knowledge, none has systematically assessed the causal relationship using a structured criterion-based framework comparable to Bradford Hill. Over the last few years, a range of issues have been discussed as being affected by the use of these molecules, including: depression and/or suicidal ideation; anxiety/sleep disturbances; eating behaviour anomalies; cognition modifications; ocular-related disorders; and substance/non-substance-related addictive behaviour. Neuropsychiatric adverse events with GLP-1 and dual GIP/GLP-1 RAs appear uncommon and generally mild. Furthermore, data on dual agonists such as tirzepatide are comparatively limited. Thus far, neuropsychiatric adverse events appear infrequent and similar in profile to those observed with GLP-1 RAs alone. No distinct neuropsychiatric safety signal specific to GIP receptor activation has been identified.

Although causality is contested and rates in spontaneous reports are low relative to prescriptions, case reports and pharmacovigilance signals, particularly concerning mood changes and suicidality, have emerged.

Indeed, several plausible pathways could explain the occurrence of neuropsychiatric outcomes: (a) rapid changes in appetite and body composition altering neuroendocrine homeostasis and psychosocial stress; (b) pre-clinically confirmed direct modulation of mesolimbic DA and other monoaminergic systems by incretin-related signaling; and (c) systemic effects such as nausea, sleep disruption or nutritional changes that secondarily influence mood.

Conversely, however, weight loss itself frequently improves depressive symptoms, self-esteem and certain metabolic contributors to cognition. Furthermore, some patients even report improved mood; anxiety; and reductions in addictive behaviour after GLP-1/GIP agonist therapy. Overall, systematic and narrative reviews find both improvements and neutral effects in cognition and mood across populations.

One would then tentatively suggest that there may be significant individual differences, possibly relating to the index client’s psychobiological vulnerability and previous psychiatric history, in response to GLP-1 and GLP-1/GIP agonist therapy administration. Hence, clinicians should monitor patients with pre-existing psychiatric conditions, assess them for new-onset depressive or anxiety symptoms, and provide appropriate follow-up.

Applying the BH criteria suggests that the relationship between GLP-1 receptor agonists and ocular complications, particularly diabetic retinopathy, may reflect an indirect effect mediated by rapid glycemic improvement rather than direct retinal toxicity. It is a reason of concern, however, that recent studies from our group [63] identified some retinopathy-related disorders associated with both semaglutide and liraglutide even in non-diabetic patients. While temporality, plausibility, and analogy support a causal interpretation, limitations in strength, specificity, and consistency of evidence indicate that further longitudinal and mechanistic studies are needed.

### Clinical considerations

Taken together, the available evidence suggests a possible association between GLP-1 receptor agonists and mood-related outcomes, but causality and directionality remain uncertain”. At present, large, long-term, adequately powered studies with systematic psychiatric assessments to clarify causality, identify at-risk subgroups, and better characterize the neuropsychiatric safety profile of this drug class are lacking. Accordingly, a few practical considerations and recommendations could be considered before starting either a GLP-1 RA or a dual GIP/GLP-1 agonist therapy.

1. *Baseline psychiatric screening*. Before initiating GLP-1 or dual GIP/GLP-1 RAs, a screening for active mood disorders, eating disorders, suicidal ideation, substance use and unstable psychiatric illnesses should be carried out;
2. *Full discussion of side effects*. A discussion about common adverse effects (nausea, vomiting, constipation, sleep disturbance) and the currently uncertain but actively studied links to mood and suicidality should occur, whilst emphasizing that large trials are ongoing;
3. *Monitoring plan*. Checking mood and suicidality at early titration visits (weeks 2–12) and periodically thereafter is advisable. For patients with a psychiatric history, closer follow-ups may be needed;
4. *Manage adverse effects proactively*. Gastrointestinal adverse effects are the most frequent cause of discontinuation; anticipatory counseling and slow titration can mitigate these. Sleep and appetite changes should be addressed and discussed; if new or worsening depression/anxiety emerges, a re-evaluation of the risk–benefit ratio should take place;
5. *Special populations*. Adolescents, individuals with severe eating disorders, or those with active suicidal ideation may require multidisciplinary risk assessment before use.

#### Research gaps and priorities

GLP-1 RAs may be effective in treating overeating/obesity, and possibly for both treatment-resistant depression and drug/alcohol use disorders. These examples reflect a transdiagnostic pharmacological profile, arguably supporting the construct of an ‘Addiction Spectrum Disorder’ (ASD; [35, 73]), a dimensional model recognizing the shared pathophysiological mechanisms underpinning seemingly disparate addictive behaviour. Importantly, the therapeutic potential of these agents must be weighed against emerging concerns regarding their non-medical use or abuse liability in vulnerable subjects [7].

Indeed, one could argue that some pharmacological/neurobiological complexities should be better understood. In fact, some studies seem to suggest a *blunted* DA response in GLP-1 and GIP/GLP-1 RAs medicated subjects when ingesting food. One could, however, argue if the food, medication-related, blunted response is enough to explain such very significant levels of body weight decrease being observed. Is it possible that, whilst on GLP-1/GIP RAs, the DA reward pathway reaches an earlier ‘satisfaction/fulfillment’? In line with this, it has been suggested that semaglutide and similar drugs may indeed promote satiety [81], because GLP-1 RA show pro-DAergic efficacy [31, 81] and can hence modulate reward [13].

From this point of view, one could conceive ASD (overeaters; alcoholics; drug abusers) subjects as individuals being chronically affected by a low/dysregulated central DA tone. Hence, they would be attracted to ingesting large levels of conventional/non-conventional reinforcers.

Indeed, large randomized trials rarely include comprehensive psychiatric phenotyping. Hence, prospective, pre-specified psychiatric endpoints, causal studies linking incretin multi-agonists to neuropsychiatric outcomes should be promoted.

Longitudinal pharmacovigilance integrating prescribing denominators, electronic health records and validated psychiatric outcomes (suicidality, new-onset mood disorders) to quantify rare adverse events with adequate precision should be considered as well [46].

### Limitations

The Bradford Hill criteria are widely used and provide a structured heuristic framework for causal inference; however, they have well-recognized conceptual and practical limitations. Causality may exist even when not all criteria are satisfied, and conversely, fulfillment of several criteria does not by itself establish causation. Moreover, a number of criteria, including strength and consistency, require subjective judgment, which may introduce interpretive bias. The biological plausibility criterion is also inherently dependent on the current state of scientific knowledge, which may be incomplete or evolving. In addition, the Bradford Hill criteria cannot substitute for well-designed longitudinal studies, randomized trials, or robust causal-inference methods that address temporality, confounding, and bias, more directly. Accordingly, the Bradford Hill criteria should be viewed as a useful framework for causal reasoning rather than a definitive test of causality.

## Conclusions

GLP-1 and dual GIP/GLP-1 receptor agonists’ central and peripheral pharmacology plausibly affects reward, appetite and mood circuits. The therapeutic potency of these agents means clinicians and psychiatrists will increasingly encounter patients taking them.

Current evidence suggests mixed effects on mental health, with many patients experiencing improvements related to weight loss, whilst some pharmacovigilance signals (mostly for GLP-1RAs) warrant cautious monitoring. Whilst GLP-1 RAs show overall considerable levels of therapeutic potential, their unresolved safety profile in patients with pre-existing psychiatric conditions necessitates a cautious clinical approach [46].

For now, clinicians should screen before initiation, monitor mood and suicidality during early treatment, reconcile psychotropic medications, and participate in, or support, systematic post-marketing surveillance. Ongoing mechanistic and prospective clinical studies are urgently needed to define benefits and rare risks precisely.

## Key References

- Cai M, Choi T, Xie Y, Al-Aly Z. Glucagon-like peptide-1 receptor agonists and risk of substance use disorders among US veterans with type 2 diabetes: cohort study. BMJ [Internet]. 2026 Mar 4;392:e086886. Available from: 10.1136/bmj-2025-086886.
  - **○ **This large cohort study of over 600,000 veterans provides the most robust clinical evidence available to date on the potential of GLP-1 RAs to reduce the incidence of substance use disorders (SUDs). The results demonstrate not only a reduced risk of new diagnoses of alcohol, opioid and nicotine dependence, but also a significant decrease in overdose and mortality among patients with pre-existing SUDs.
- Anderer S. FDA requests removal of suicidal ideation warning from GLP-1 RA medications. JAMA [Internet]. 2026 Jan 30;335(8):659. Available from: 10.1001/jama.2025.26370.
  - **○ **This represents the definitive regulatory benchmark for the psychiatric safety of the class. Based on a meta-analysis of 91 clinical trials (over 100,000 patients) and Sentinel system data on millions of users, the FDA concluded that there is no increased risk of suicidal ideation compared to placebo or other treatments, leading to the removal of the safety warnings.
- Völker KM, Prechtl BLH, Bormann NL, Choi DS. The potential role of GLP-1 receptor agonists in substance use disorders – a systematic review. Frontiers in Pharmacology [Internet]. 2026 Jan 2;16:1702448. Available from: 10.3389/fphar.2025.1702448.
  - **○ **A key systematic review of the biological plausibility of addiction treatment. It describes in detail how activation of GLP-1 receptors in the central nervous system modulates dopamine release in the nucleus accumbens, attenuating the reinforcing properties of alcohol, nicotine and drugs without suppressing basal tonic dopamine.
- Ouyang C, Zhang X, Zhang M, Miao Y, Zhu Z, Zeng Y, et al. Causal association between glucagon-like peptide-1 receptor agonists and mental disorders: insight from genetic and real-world evidence. Journal of Affective Disorders [Internet]. 2026 Feb 17;403:121452. Available from: 10.1016/j.jad.2026.121452.
  - **○ **A study of the highest methodological standard, using Mendelian randomisation to investigate genetic causality. The results rule out a causal link between GLP-1 RAs and anxiety or bipolar disorder, while demonstrating a significant impact on eating disorders, thereby supporting the concept of a transdiagnostic pharmacological profile.
- Murray M, Schifano F, Chiappini S, Corkery JM, Guirguis A. Potential eye disorders in people with and without Type 2 diabetes mellitus exposed to GLP-1 receptor agonists: an examination of the FAERS (FDA Adverse Event Reporting System) database. American Journal of Ophthalmology [Internet]. 2025 Dec 20;283:279–90. Available from:10.1016/j.ajo.2025.12.015.
  - **○ **A pivotal study that has raised a new ocular safety concern. By analysing the FAERS database, the study identified reports of retinopathy and retinal haemorrhage not only in diabetic patients but also in non-diabetic populations, suggesting that ocular damage may be a direct effect and not solely related to the rapid fall in blood glucose.
- Zhu Z, Gong R, Rodriguez V, Quach KT, Chen X, Sternson SM. Hedonic eating is controlled by dopamine neurons that oppose GLP-1R satiety. Science [Internet]. 2025b Mar 27;387(6741):eadt0773. Available from: 10.1126/science.adt0773.
  - **○ **A foundational neuroscience study published in Science that elucidates the mechanism of reward 'blunting'. It demonstrates how dopaminergic neurons modulate hedonic eating by counteracting GLP-1 satiety signals, thereby providing the scientific basis for understanding why these drugs reduce food-related pleasure.
- Arillotta D, Floresta G, Guirguis A, Corkery JM, Catalani V, Martinotti G, et al. GLP-1 Receptor Agonists and Related Mental Health Issues; Insights from a Range of Social Media Platforms Using a Mixed-Methods Approach. Brain Sciences [Internet]. 2023 Oct 24;13(11):1503. Available from: 10.3390/brainsci13111503.
  - **○ **Groundbreaking study exploring 'real-world evidence' through analysis of social media (Reddit, TikTok, YouTube). It highlights how patients' subjective experiences are polarised, with frequent reports of anxiety, insomnia and depression emerging from online discussions, underlining the importance of clinical monitoring beyond trial data.
- Sharafshah A, Lewandrowski KU, Gold MS, Fuehrlein B, Ashford JW, Thanos PK, et al. In silico Pharmacogenomic assessment of glucagon-like peptide-1 (GLP1) agonists and the Genetic Addiction Risk Score (GARS) related Pathways: implications for suicidal ideation and Substance use disorder. Current Neuropharmacology [Internet]. 2025 Jan 27;23(8):974–95. Available from: 10.2174/011570159x349579241231080602.
  - **○ **Pharmacogenomic analysis introducing the concept of a 'double-edged sword' for GLP-1 RAs. It suggests that individual genetic vulnerability, as measured by the GARS score, may determine whether a patient will benefit from the drug or whether they will be at risk of exacerbating depressive phenotypes due to reward system blockade.

## Data Availability

No datasets were generated or analysed during the current study.
